# A Novel Piecewise Cubic Hermite Interpolating Polynomial-Enhanced Convolutional Gated Recurrent Method under Multiple Sensor Feature Fusion for Tool Wear Prediction

**DOI:** 10.3390/s24041129

**Published:** 2024-02-08

**Authors:** Jigang He, Luyao Yuan, Haotian Lei, Kaixuan Wang, Yang Weng, Hongli Gao

**Affiliations:** 1 School of Mechanical Engineering, Southwest Jiaotong University, Chengdu 610031, China; hjgssg@my.swjtu.edu.cn; 2 School of Mathematics, Sichuan University, Chengdu 610065, China; yuanluyao@stu.scu.edu.cn (L.Y.); hao@stu.scu.edu.cn (H.L.); eeentity@stu.scu.edu.cn (K.W.)

**Keywords:** deep learning, cutter wear prediction, Piecewise Cubic Hermite Interpolating Polynomial (PCHIP), Convolutional Neural Network (CNN), Gated Recurrent Unit (GRU)

## Abstract

The monitoring of the lifetime of cutting tools often faces problems such as life data loss, drift, and distortion. The prediction of the lifetime in this situation is greatly compromised with respect to the accuracy. The recent rise of deep learning, such as Gated Recurrent Unit Units (GRUs), Hidden Markov Models (HMMs), Convolutional Neural Networks (CNNs), Recurrent Neural Networks (RNNs), Attention networks, and Transformers, has dramatically improved the data problems in tool lifetime prediction, substantially enhancing the accuracy of tool wear prediction. In this paper, we introduce a novel approach known as PCHIP-Enhanced ConvGRU (PECG), which leverages multiple—feature fusion for tool wear prediction. When compared to traditional models such as CNNs, the CNN Block, and GRUs, our method consistently outperformed them across all key performance metrics, with a primary focus on the accuracy. PECG addresses the challenge of missing tool wear measurement data in relation to sensor data. By employing PCHIP interpolation to fill in the gaps in the wear values, we have developed a model that combines the strengths of both CNNs and GRUs with data augmentation. The experimental results demonstrate that our proposed method achieved an exceptional relative accuracy of 0.8522, while also exhibiting a Pearson’s Correlation Coefficient (PCC) exceeding 0.95. This innovative approach not only predicts tool wear with remarkable precision, but also offers enhanced stability.

## 1. Introduction

In industrial scenarios, when equipment is used for processing, the lifetime and maintenance of the equipment are factors that must be considered. For example, in the context of Computerized Numerical Control (CNC) machine processing, the maintenance of the tool’s lifetime is particularly important and has the highest priority. This is due to the necessity for more frequent replacement of severely worn tools, thereby increasing the downtime and maintenance expenses along the production line. Meanwhile, tool wear affects the quality of machined parts, leading to uneven machined surfaces, dimensional inaccuracies, and potential damage to the workpiece. Severely worn tools can even pose a safety hazard to the working environment and the operators. In essence, the prediction of tool wear not only contributes to heightened production efficiency, cost control, and product quality assurance, but also aligns with the trend towards intelligent manufacturing. This progression fosters the development of the manufacturing industry in a direction that is more advanced, sustainable, and intelligent.

The state of cutting tools has an important impact on production efficiency and surface processing quality. Therefore, online monitoring and real-time prediction of tool wear are of great significance, and they also have become the most discussed and researched hot topic in the mechanical field. Over the years, researchers have explored various methodologies and techniques to predict tool wear, aiming to enhance productivity, optimize the tool lifetime, and minimize machine downtime [1,2,3]. The earliest monitoring of cutting tool conditions started with a single variable, known as direct measurement, and gradually evolved to fewer variables, known as indirect measurement. For instance, the optical image method was the earliest traditional method applied to tool wear monitoring [4,5]; it uses the reflectance of the worn surface to evaluate the wear of the tool. Contact resistance measurement is performed using electrical resistance and the radioactive elements [6]. However, a single signal has its own drawbacks. While some processes are too complicated, some are not suitable for large workpieces, some will be affected by noise, some signal acquisition will be delayed, and some are expensive (acoustic emission monitoring of the equipment). Therefore, multiple sensor signals are widely used to monitor tool wear. The incorporation of multi-signal conditions, which involves monitoring and analyzing a wide range of parameters including vibration, temperature, acoustic emission, and cutting force, among others, has provided a more-comprehensive understanding of the tool’s behavior during machining processes. By considering a multitude of signals, engineers can gain a more nuanced insight into the complex interactions that affect tool wear and failure. This not only leads to more accurate predictions, but also enables proactive maintenance and optimization strategies. Multiple sensor signals mean multiple features, and their fusion starts to become the key [7,8].

In recent years, with the popularity of machine learning and deep learning, new directions have opened up for research on cutting tools, and numerous related studies have sprung up using methods such as Artificial Neural Networks (ANNs) [9,10], Support Vector Machines (SVMs) [11,12,13], the Hidden Markov Model (HMM) [14,15,16,17], Gaussian Process Regression (GPR), etc. [18,19]. With the rise of deep learning, these types of methods have advanced to a new level [20]. In the contemporary landscape of modern manufacturing, the incorporation of multi-signal conditions and the utilization of deep learning in tool lifetime prediction are essential for fostering efficiency, reliability, and competitiveness. Deep learning methods have shown significant promise in tool wear prediction for machining processes due to their ability to automatically learn complex patterns and relationships from large datasets [21]. They have the potential to outperform traditional analytical and empirical models by capturing intricate nonlinearities in the machining process. Convolutional Neural Networks (CNNs), Recurrent Neural Networks (RNNs), Attention networks, and so on, have skyrocketed in the mechanical field [22,23,24]. These models, primarily known for their remarkable achievements in areas such as computer vision and language translation, have found substantial relevance in the realm of mechanical production as well, underpinning the evolution of smart manufacturing.

It is important to note that deep learning methods require large, labeled datasets for effective training, which can be a challenge in some machining scenarios. Data augmentation is a technique widely used in machine learning to artificially increase the size of a training dataset by applying various transformations to the original data. This helps improve the model’s generalization and robustness. When it comes to machine learning for tool wear prediction, data augmentation can be particularly beneficial in enhancing the model’s ability to recognize patterns associated with different states of tool wear. Usually, in the collection of data on tool wear, only the values of the sensor signals (such as the cutting force, vibration, acoustic emission, and current) are collected, but the value of the tool wear is not measured. The main reason is that the signal acquisition sensors are attached to, for example, a CNC machine tool, so they can collect the data at a relatively high frequency, and the amount of wear of the tool is measured after the tool has been used for a constant interval, so the frequency of the obtained data is much lower. Therefore, we need to use data augmentation methods to improve the data availability. Data augmentation is a commonly used technique in machine learning, involving the transformation and expansion of training data to enhance their diversity and richness. It improves the model’s generalization capability, robustness, and accuracy. There are multiple methods available for data augmentation, including random erasing [25], data interpolation [26], and so on. By addressing issues such as overfitting, imbalanced data, missing data, and limited samples, data augmentation effectively enhances the performance and reliability of machine learning models [27].

In the context of tool wear prediction with deep learning, data augmentation refers to the technique of artificially increasing the size and diversity of the training dataset by applying various transformations to the original sensor data collected during machining processes, with the goal of enhancing the generalization and robustness of the deep learning model by exposing it to a wider range of variations and scenarios that may be encountered in real-world tool wear conditions. For tool wear prediction, the input data often consist of sensor readings, such as vibration signals, acoustic signals, current signals, or other sensor data collected during the cutting or machining process. In such operating conditions, tool wear stages, or machining scenarios simulated by introducing variations to sensor data using a data augmentation method, the Piecewise Cubic Hermite Interpolating Polynomial (PCHIP) interpolation method can be used to obtain the uniformly spaced interpolation data series. In a modified grey model proposed by Wang F et al. to predict the RUL of rolling bearings based on vibration data, the PCHIP method was used to process the original data, and this method managed to maintain the trend characteristics of the original signal while improving the reliability of RUL prediction results [28].

The PCHIP-Enhanced ConvGRU (PECG) model we introduced adeptly merges CNN and GRU networks to effectively capture the time series characteristics of the tool wear data. In our study, thanks to the National Natural Science Foundation of China, we were able utilize real industrial tool data versus synthetic datasets, which lends credence to the model’s wear prediction results. In real scenarios, we employed the PCHIP method to interpolate and supplement the wear data, aimed at addressing incomplete tool wear data due to rapid sensor data acquisition. This approach alleviates the issue of high-dimensional but insufficient measurement data obtained from sensor-based tool wear measurements. Notably, PCHIP interpolation substantially elevated the relative prediction accuracy of the model from 0.8005 to 0.8522. Our methodology further involves the extraction of local features via the CNN layer, leveraging the resulting feature map as input for the GRU encoder to capture temporal dependencies. While fully exploiting the time series information processing capabilities of GRU, PECG effectively harnesses the spatial feature learning process of CNN, thereby organically combining and maximizing the strengths of both.

In summary, based on the research trend of multi-feature fusion in the industry, and the advantages of deep learning to mine data, a new PECG method under multiple feature fusion for tool wear prediction has been developed. Our proposed method has the following contributions:

- By employing the Piecewise Cubic Hermite Interpolating Polynomial method in tandem with an understanding of the patterns associated with missing tool wear data, we successfully interpolated and completed the wear data. This approach effectively resolves the challenge posed by high-dimensional tool wear measurement data collected by sensors, a scenario often characterized by relatively insufficient measurement data.
- We extract local features through the CNN layer, leveraging the feature map as input for the GRU encoder to capture temporal dependencies. The PECG model effectively harnesses the spatial feature learning capacity of CNN while fully optimizing the time series data processing abilities of GRU. This results in the seamless integration and maximization of the strengths of both models, making it particularly well-suited for processing data characterized by both time series and spatial features.
- These two aspects are combined to form a comprehensive PECG method.

The remainder of this paper is organized as follows. Section 2 introduces the data interpolation method, PCHIP. Section 3 describes the proposed wear prediction model in detail. In Section 4, we conduct experimental studies to compare the proposed model with other methods and confirm its superiority. Section 5 provides conclusions. The abbreviations are listed at the end of this paper.

## 2. PCHIP Interpolation Method

In the data acquisition process, the varying methods of acquiring data have led to a significantly higher volume of sensor data compared to wear data, resulting in a lack of corresponding wear data for certain sensor readings. Consequently, there are missing values within the wear data. Previous approaches involved the deletion of sensor data lacking corresponding wear data, inadvertently discarding valuable information inherent in the sensor data. To address this issue, we have introduced the PCHIP interpolation method to substitute the missing wear data. Through this method, we establish a one-to-one correspondence between sensor data and tool wear data, ensuring the maximization of information encapsulated within the sensor data. This approach enables us to fully leverage the information gleaned from sensor data while circumventing the loss of valuable insights.

There are many interpolation methods. Among them, the simplest method is to define a piecewise linear function between each number of points. The linear method is fast and easy to implement, but linear interpolation does not produce a smooth curve. To solve this problem, a higher-order polynomial can be chosen between each pair of data points, and we can specify the gradient of this polynomial to ensure that the overall approximation function is continuous and has continuous derivatives. Cubic spline interpolation resolves sudden changes in gradients in the case of linear interpolation. But this also introduces a problem that the interpolation may be outside the range of our data point values, which can lead to overshooting issues.

We use Piecewise Cubic Hermite Interpolating Polynomial (PCHIP) to avoid the above two problems. The cubic Hermite polynomial is defined as follows:

(1) $$\begin{array}{cc} p(t) & =h_{00}\left(t\right)p_{0}+h_{10}\left(t\right)\left(x_{k+1}-x_{k}\right)m_{0}+h_{01}\left(t\right)p_{1}+h_{11}\left(t\right)\left(x_{k+1}-x_{k}\right)m_{1} \end{array}$$

where $h_{00},h_{10},h_{01},h_{11}$ are Hermite basis functions. PCHIP interpolates using a piecewise cubic polynomial P(x) with these properties:

- On each subinterval $x_{k}\;\leq \;x\;\leq \;x_{\left(k+1\right)}$, the polynomial P(x) is a cubic Hermite interpolating polynomial for the given data points with specified derivatives at the interpolation points.
- P(x) interpolates *y*, that is, $p\left(x_{j}\right)=y_{j}$, and the first derivative $\frac{dp}{dx}$ is continuous. The second derivative $\frac{d^{2}p}{dx^{2}}$ is probably not continuous, so jumps at $x_{j}$ are possible.
- The cubic interpolant P(x) is shape-preserving. The slopes at $x_{j}$ are chosen in such a way that P(x) preserves the shape of the data and respects monotonicity. Therefore, on intervals where the data are monotonic, so is P(x), and at points where the data have a local extremum, so does P(x).

These properties of the piecewise cubic polynomial maintain the monotonicity of the points on the interpolation curve [29]. They solve the problem of overshoot and the curve of the interpolation result is smooth at the same time.

## 3. Model Construction

Data-driven methods predict tool wear using predictive models trained by machine learning or pattern recognition algorithms [30]. When dealing with data-driven works, deep learning is able to learn from large amounts of data and identify subtle patterns and relationships between tool wear value and sensor data.

**Figure 1:**
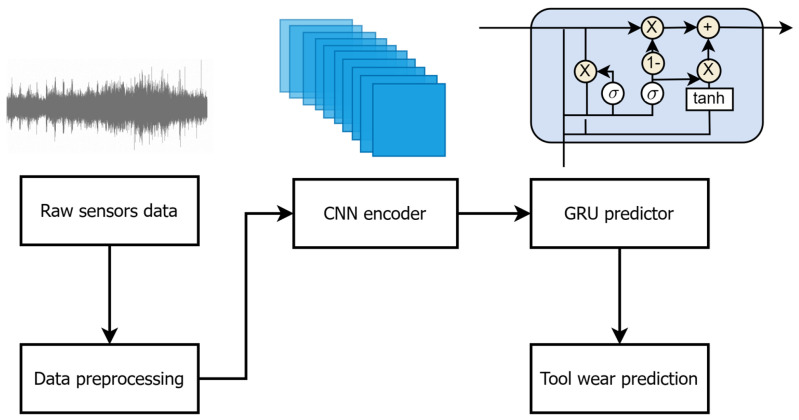
The framework of PECG method.

As shown in Figure 1, the proposed PECG mainly includes two stages: data preprocessing and model construction. After the data preprocessing, we successfully resolved the problem of missing wear data by employing the PCHIP interpolation technique. The processed data were subsequently utilized to train the proposed model. The details of the model construction are illustrated below.

### 3.1. Convolutional Neural Network

CNNs are primarily used for image classification tasks and have become dominant in various computer vision tasks, but they can also be used for regression problems. A CNN has five basic layers: convolutional layer, pooling layer, activation layer, fully connected layer, and dropout layer. In this paper, we use a CNN as a feature extractor and pass the features to a GRU. In that case, the CNN in our method incorporates a convolutional layer followed by batch normalization and an activation layer. The equation for this process is as follows:

(2) $$\begin{array}{cc} c_{ik} & =\mathrm{ReLU}\left(W_{k}*x_{i}+b_{k}\right) \end{array}$$

where $W_{k}$ indicates the convolutional filter, * denotes the convolution operation, $b_{k}$ is the bias, and the activation function is ReLU. Here, $c_{ik}$ represents the encoding result, which is the extracted feature we use in the followed GRU.

### 3.2. Gated Recurrent Unit

The Gated Recurrent Unit (GRU) is a type of Recurrent Neural Network (RNN) architecture that has gained popularity in recent years due to its ability to model sequential data with greater efficiency and accuracy. In this paper, we use a GRU model after the CNN to obtain wear predictions. In a GRU model, there are two gates: an update gate and a reset gate. The update gate determines how much of the previous hidden state should be retained and how much of the current input should be added to the new hidden state, while the reset gate controls how much of the previous hidden state should be ignored. These gating mechanisms allow the GRU model to selectively remember or forget information from the past. Equations for this process are as follows:

(3) $$\begin{array}{cc} r_{t} & =\sigma \left(W_{ir}x_{t}+b_{ir}+W_{hr}h_{\left(t-1\right)}+b_{hr}\right) \end{array}$$

(4) $$\begin{array}{cc} z_{t} & =\sigma \left(W_{iz}x_{t}+b_{iz}+W_{hz}h_{\left(t-1\right)}+b_{hz}\right) \end{array}$$

(5) $$\begin{array}{cc} n_{t} & =\tanh\left(W_{in}x_{t}+b_{in}\;+\;r_{t}*\left(W_{hn}h_{\left(t-1\right)}+b_{hn}\right)\right) \end{array}$$

(6) $$\begin{array}{cc} h_{t} & \;=\;\left(1-z_{t}\right)*n_{t}+z_{t}*h_{\left(t-1\right)} \end{array}$$

where $h_{t}$ is the hidden state at time *t*, $x_{t}$ is the input at time *t*, $h_{\left(t-1\right)}$ is the hidden state of the layer at time t−1 or the initial hidden state at time *o*, and $r_{t},z_{t},n_{t}$ are the reset, update, and new gates, respectively. σ is the sigmoid function, and ∗ is the Hadamard product. Then, the result of the hidden state is imported to a fully connected layer and the output is the wear prediction result.

### 3.3. Model Framework

**Figure 2:**
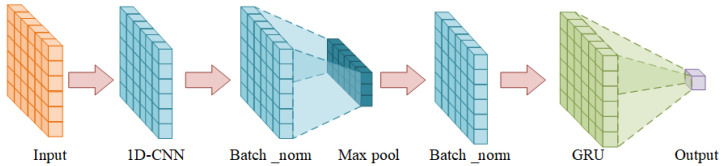
Structure of PECG.

The framework of PECG is illustrated in Figure 2. And details of our model structure are shown in Table 1. In a CNN, the convolutional layers are used to extract features from the input data. CNN has the ability to capture complex patterns and relationships in the input data. In that case, after analyzing the data, which have high-dimensional sensor data as input, we first use a one one-dimensional ten-layer CNN as an encoder to extract features and reduce the dimensionality of the data. The output of the CNN encoder is then imported to a GRU. Finally, the wear prediction is completed through a fully connected layer.

## 4. Experiment and Result

### 4.1. Experimental Conditions

**Figure 3:**
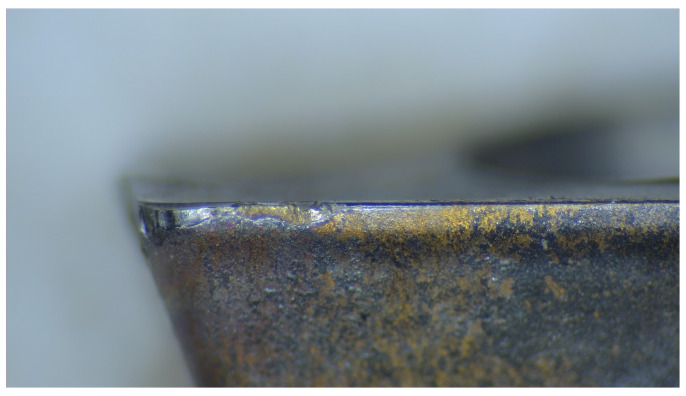
Real situation of tool wear on machine tools.

We utilized tool data acquired with support from the National Natural Science Foundation of China, gathered from real industrial settings, as input for the model, rather than relying on virtual datasets available through networks. This approach significantly enhances the credibility of the wear prediction results. The milling cutter under consideration is the APMT1135 carbide cutter, a product of Duracarb. Its fundamental parameters include a tool tip angle of 85 degrees, a blade relief angle of 11 degrees, a blade length of 11 mm, a thickness of 3.5 mm, an inscribed circle diameter of 6.35 mm, and a maximum cutting depth of 9 mm. Figure 3 depicts the actual state of tool wear observed on the machinery.

**Figure 4:**
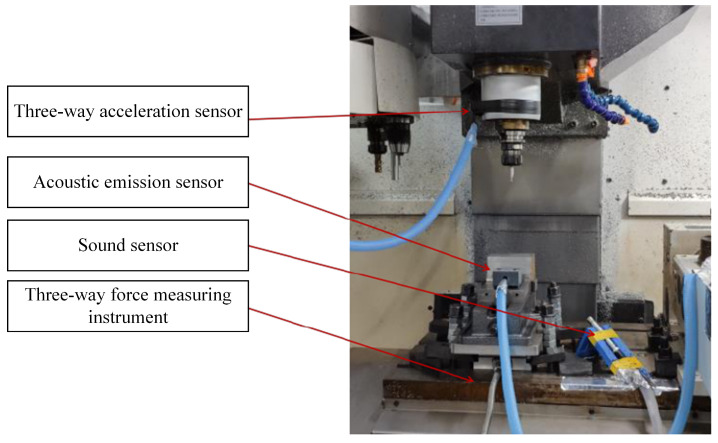
Sensor layout a. PHI Mechatronics Technology Laboratory, Chengdu, China.

**Figure 5:**
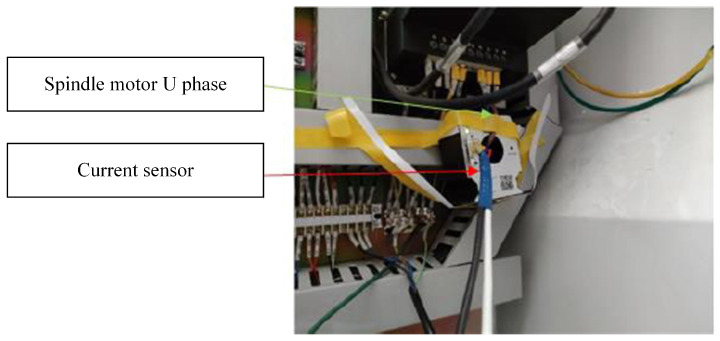
Sensor layout b. PHI Mechatronics Technology Laboratory, Chengdu, China.

**Figure 6:**
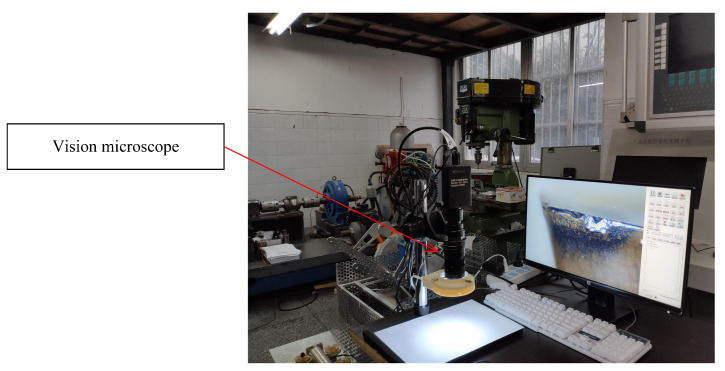
Wear amount collection. PHI Mechatronics Technology Laboratory, Chengdu, China.

There are four types of sensor signal collected during the cutting process: three-way force signals, three-way vibration signals, acoustic signals, and current signals. The sensor devices are shown in Figure 4 and Figure 5. After one cutting path is completed (or after multiple cutting paths are completed), the experimental tool is removed and the wear amount is measured through a visual microscope. The measurement process of tool wear amount is illustrated in Figure 6.

The vibration signal is captured using the PCB365A15 three-way acceleration sensor, while the cutting force sensor employed is the KISTLER 9257B three-way load cell. Additionally, the setup includes the Bruel Kjaer’s 4966-H-041 acoustic sensor, the PAC-WD acoustic emission sensor, and the POLARISMMI200B (current model: CSA201-P030T01) current sensor. These diverse datasets have been instrumental in supporting the publication of several articles on milling cutter life prediction and intelligent operation in esteemed journals [24,31,32,33,34]. Furthermore, these datasets represent the lifecycle patterns observed in carbide cutters.

### 4.2. Dataset

We carry out basic data cleaning for the collected wear data, which is divided into three parts: standardization, partial correction, and elimination. When we collect these eight types of data, we first standardize them:

(7) $$x_{s}=\frac{x_{i}-\bar{x}}{\sigma }$$

where $x_{s}$ is the standardized data. $\bar{x}$ represents the mean of the data. σ represents the standard deviation of the data.

The reason for this is obvious: to scale the data so that they fall into a small, specific interval. Standardization solves the problem of small difference in working conditions by scaling according to variance. It is often used in some comparison and evaluation index processing to remove the unit restriction of the data and convert it into a dimensionless pure value, so that indicators of different units or magnitudes can be compared and weighted.

**Figure 7:**
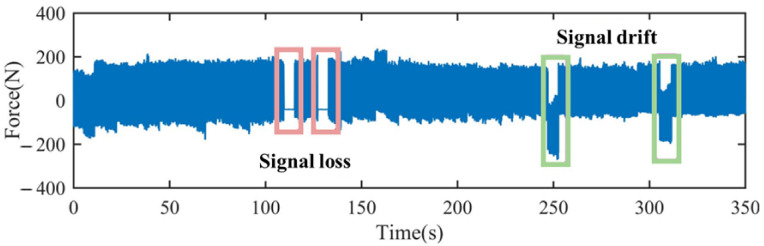
Signal loss and signal drift.

The signal drift of the data is shown in Figure 7. The obvious missing and drifting parts of life data monitoring are shown in the red and green boxes, respectively. We use Exponential Moving Average (EMA) to bring significantly drifting segments of the data back into the normal range:

(8) $$v_{t}=\beta \cdot v_{t-1}+\left(1-\beta \right)\cdot \theta_{t}$$

where $v_{t}$ represents the average value of the first t bars ($v_{0}=0$), β is the weighting value (generally set to 0.9–0.999), and $\theta_{t}$ is the standardized data.

Furthermore, we eliminate obviously abnormal data [35]:

(9) $$\delta =\sqrt{\frac{1}{m-1}\sum_{k=1}^{m}{\left(S_{K}-\frac{\sum_{k=1}^{m}S_{K}}{m}\right)}^{2}}$$

where δ is the abnormal data. The pseudocodes for describing the processes to display the data that cannot be used directly and need to be eliminated are shown in the following Algorithm 1.

Our dataset includes data collected from 28 milling cutters under eight different cutting conditions. The details of the cutting conditions of the cutters are shown in Table 2. In this table, Cm_n is the sign of a cutter, which means no. n cutter under condition m. The signals of cutters C4_3 and C7_9 are used as the test data, and the rest of the cutters are used as the training set. Deep learning makes it possible to involve all signals, making wear prediction accurate and efficient. Single sensor signals often have their own limitations, but deep learning possesses powerful feature learning capabilities. By using a multi-signal variable matrix for prediction, it is possible to extract rich information from multiple features, effectively avoiding the limitations associated with relying on a single feature. We use a total of eight variables: current, force (three directions, x,y,z), sound, and vibration (three directions, x,y,z) to predict the wear process.
**Algorithm 1** Signal_Segment ($Sig_{org}$, $L_{w}$, $d_{w}$)**Inputs**:$Sig_{org}$—original time-domain signal$L_{w}$—width of sliding window$d_{w}$—moving step length of sliding window**Outputs**:$Mat_{window}$—data window matrix 1: **Calculate** cl2: **Initialize** $Mat_{window}$3: **for** i=1 to cl do4: **if** i=15: Assign the data from 1 to $L_{w}$ in $Sig_{org}$ to the *i*th column of the $Mat_{window}$.6: **else if** i!=cl7: Assign the data who are located from the $\left(i*d_{w}+1\right)$th to the $\left(i*d_{w}+1+L_{w}\right)$th in $Sig_{org}$ to the *i*th column of $Mat_{window}$.8: **else**9: Assign the data who are located from the $\left(i*d_{w}+1\right)$th to the end of $Sig_{org}$ to the *i*th column of $Mat_{window}$ and replace the Null in the *i*th column with 0.11: **End if**12: **End for**

**Figure 8:**
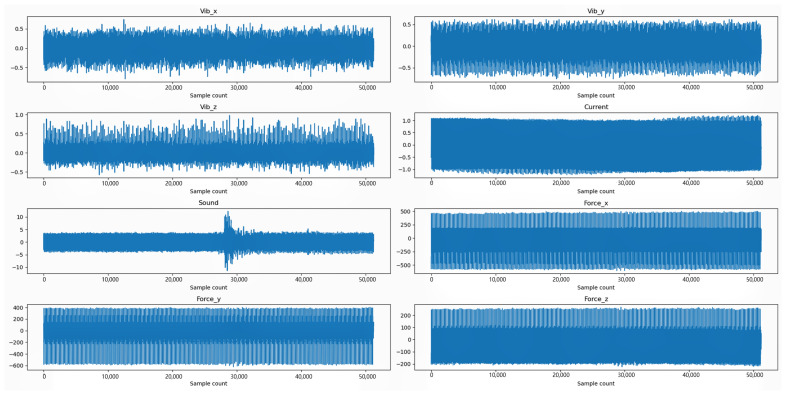
Monitoring signal data of C4_3.

Figure 8 shows the used sensor signal of cutter C4_3, including the vibration signal, current signal, sound signal, and force signal. It can be found that there is no clear trend in the data. In that case, we employ our model to extract more information.

**Figure 9:**
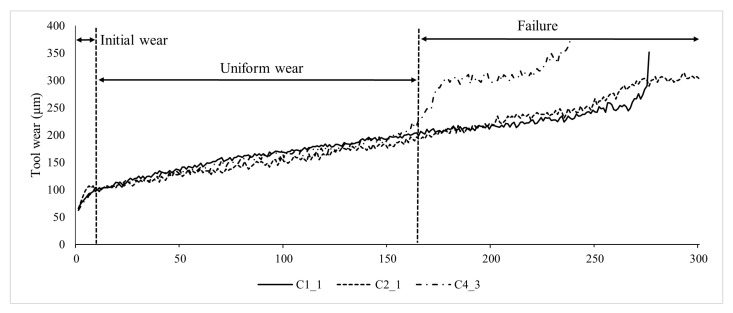
Wear stage of C1_1, C2_1, C4_3.

The wear data of the tool indicate that the process of tool wear can be divided into three stages: the initial wear stage, the normal wear stage, and the rapid wear stage. First, there is the initial wear stage. Due to the regrinding of the tool, the cutting edge and tool surface are not smooth enough, resulting in a small actual contact area between the back surface of the tool and the cutting surface, but with high pressure. Therefore, the wear is rapid but for a short period of time. Next is the normal wear stage. After the initial wear, the contact area between the back surface of the tool and the workpiece increases, and the pressure per unit area decreases gradually. The micro-rough surface of the back surface of the tool is smoothed out, resulting in a slower wear rate. This stage represents the tool’s effective working phase. Finally, there is the rapid wear stage. When the amount of tool wear reaches a certain limit, the cutting force and cutting temperature increase dramatically, leading to an accelerated tool wear rate until the tool loses its cutting ability. This stage is referred to as the rapid wear stage. The tool must be replaced before entering the rapid wear stage. As shown in Figure 9, three tools, C1_1, C2_1, and C4_3, demonstrate the three stages of wear. It can be observed that initially, the tool wear rapidly increases within a short period, then the growth rate slows down until the rapid wear stage, where the wear value starts to increase rapidly again.

**Figure 10:**
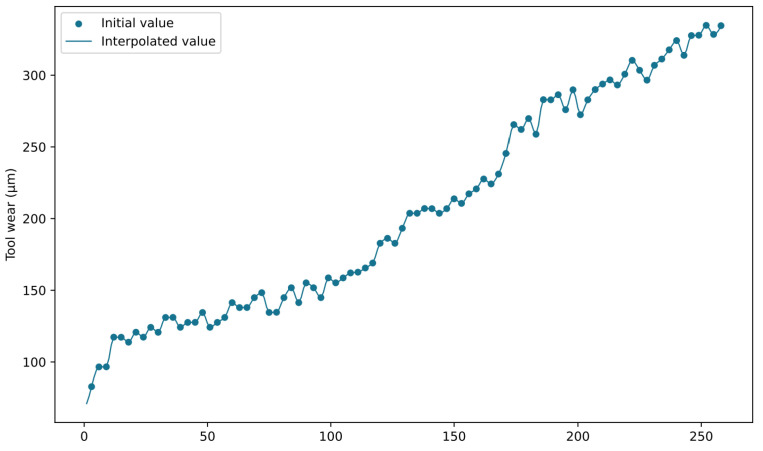
Interpolation results of C6_1.

**Figure 11:**
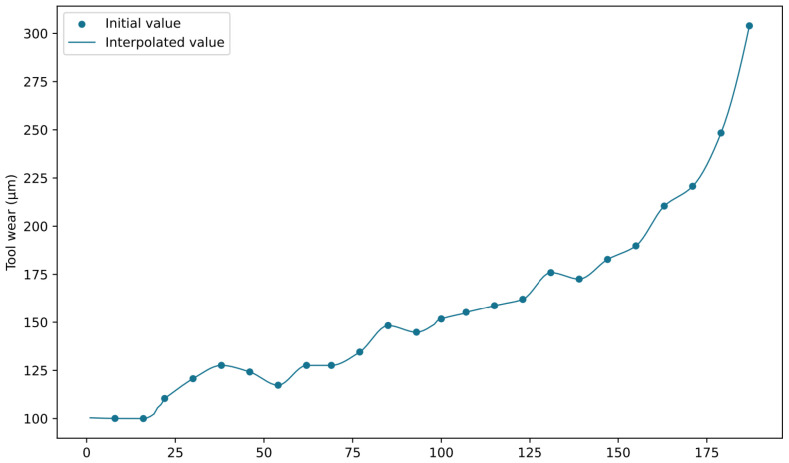
Interpolation results of C8_1.

After data cleaning, we utilized the PCHIP method to conduct data augmentation on the missing portions of tool wear in the dataset, aiming to expand the application of information within the dataset. The interpolation results are depicted in Figure 10 and Figure 11. Figure 10 showcases the interpolated outcome for C6_1, and Figure 11 displays the interpolated outcome for C8_1. The PCHIP interpolation method significantly resolves the problem of missing wear data, enabling the utilization of rich sensor feature information associated with the previously absent wear values.

We evaluate the performance of interpolation methods by choosing the data which do not have missing wear values and part of the true wear values, comparing the true wear values with the interpolation values. We compare PCHIP with three other common approaches—cubic spline, spline and linear by PCC, MAE, RMSE, MAPE, and standard deviation. The result is shown in Table 3. It shows that the result of PCHIP is the best among all methods below. According to the results below, it can be seen that PCHIP interpolation is better than other interpolation methods in PCC, MAE, RMSE, and standard deviation. PCHIP has the best interpolation performance. Therefore, we choose the PCHIP interpolation method to perform wear data interpolation work.

### 4.3. Prediction Results and Comparison

**Figure 12:**
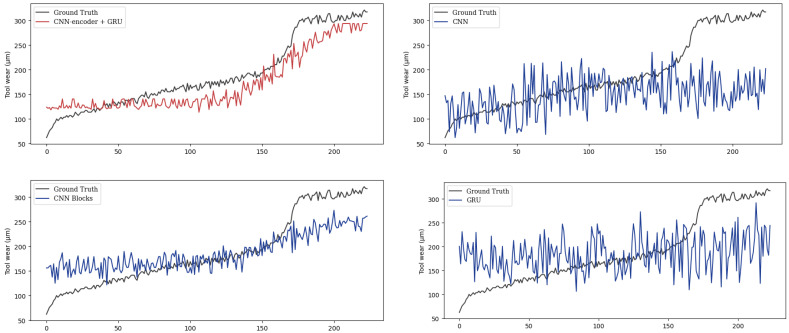
Tool wear prediction results of C4_3.

**Figure 13:**
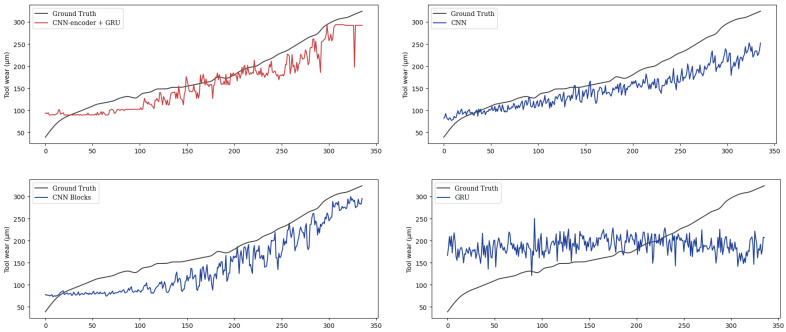
Tool wear prediction results of C7_9.

To demonstrate the effectiveness of the proposed methods, we compare it with the other three methods on the same test dataset. We represent the proposed method as PECG. And the other three models are denoted as CNN, CNN Blocks, and GRU. The CNN method only uses a one-dimensional CNN. And the CNN Blocks method contains a configurable number of convolutional blocks. The GRU method only includes a GRU model. The tool wear prediction result of the cutters C4_3 and C7_9 using the four different models is shown in Figure 12 and Figure 13. It can be seen that our combination of CNN and GRU is superior to the model which only uses CNN or RNN. It shows that PECG can effectively extract features from high-dimensional data and as we can see, it can more accurately capture the underlying trend in the data. The CNN Blocks model captures the trend at first, but when the wear value suddenly changes, it fails to complete the prediction. This result also demonstrates that our model produces more robust and less volatile predictions compared to the other models.

To further quantify the effectiveness of our proposed model, we introduced five key evaluation metrics, including Pearson Correlation Coefficient (PCC), Mean Absolute Error (MAE), Root Mean Squared Error (RMSE), Standard Deviation, and Relative Accuracy. These metrics were calculated using the same test set to assess the performance of our model. The equations for these metrics are as follows:

- Pearson Correlation Coefficient (PCC)
  PCC measures the linear correlation between predicted and actual values, ranging from −1 to 1.
  (10) $$\mathrm{PCC}=\frac{n\sum_{i=1}^{n}x_{i}y_{i}-\sum_{i=1}^{n}x_{i}\sum_{i=1}^{n}y_{i}}{\sqrt{\left[n\sum_{i=1}^{n}x_{i}^{2}-{\left(\sum_{i=1}^{n}x_{i}\right)}^{2}\right]\left[n\sum_{i=1}^{n}y_{i}^{2}-{\left(\sum_{i=1}^{n}y_{i}\right)}^{2}\right]}}$$
- Mean Absolute Error (MAE)
  MAE measures the average absolute difference between predicted and actual values.
  (11) $$\mathrm{MAE}=\frac{1}{n}\sum_{i=1}^{n}\left|y_{i}-\hat{y_{i}}\right|$$
- Root Mean Squared Error (RMSE)
  RMSE measures the square root of the average squared difference between predicted and actual values.
  (12) $$\mathrm{RMSE}=\sqrt{\frac{1}{n}\sum_{i=1}^{n}{\left(y_{i}-{\hat{y}}_{i}\right)}^{2}}$$
- Standard Deviation
  The standard deviation of errors is an indicator of the robustness of a model. A lower standard deviation signifies a higher degree of stability of the prediction performance.
  (13) $$\mathrm{SD}=\sqrt{\frac{\sum_{i=1}^{n}{\left(e_{i}-\bar{e}\right)}^{2}}{n}}$$
- Relative Accuracy
  Relative accuracy is a measure of the error or difference between a measured or calculated value and the true value of a quantity, ranging from 0 to 1.
  (14) $$\mathrm{RA}=1-\frac{1}{n}\frac{\sum_{i=1}^{n}\left|y_{i}-{\hat{y}}_{i}\right|}{\sum_{i=1}^{n}y_{i}}$$

**Figure 14:**
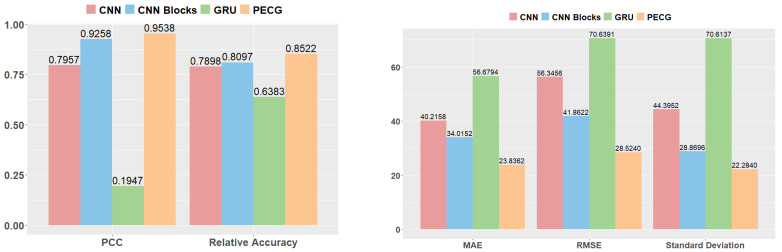
Tool wear performance estimation results of four networks.

The metrics of the four models are illustrated in Table 4 and Figure 14. Among these models, PECG performs the best in all metrics. GRU performs the worst due to poor feature extraction. The PCC of GRU is quite low, at only 0.1947, and the relative accuracy is 0.6383, which is terrible, too. The results shows that the single GRU is not suitable for performing regression. It can be seen that the CNN Blocks method performs better than CNN. Its PCC is 16.4% higher than CNN. Nevertheless, its performance can be improved. When we combine GRU with CNN Blocks, PECG outperforms those of all other models tested, providing strong evidence for its superior performance. The PCC of PECG is 0.9538, which highlights the strong correlation between predicted and actual wear values. Its standard deviation is about half of CNN. As a result of the integration of CNN Blocks and GRU, the relative accuracy of PECG is 0.8522, which is superior to the other three models. The design of PECG is less complex than the time–space attention model [24], while delivering superior performance outcomes. The relative accuracy of the time–space attention model is 0.7890. In comparison, PECG exhibits a relative accuracy that is 8% higher.

**Figure 15:**
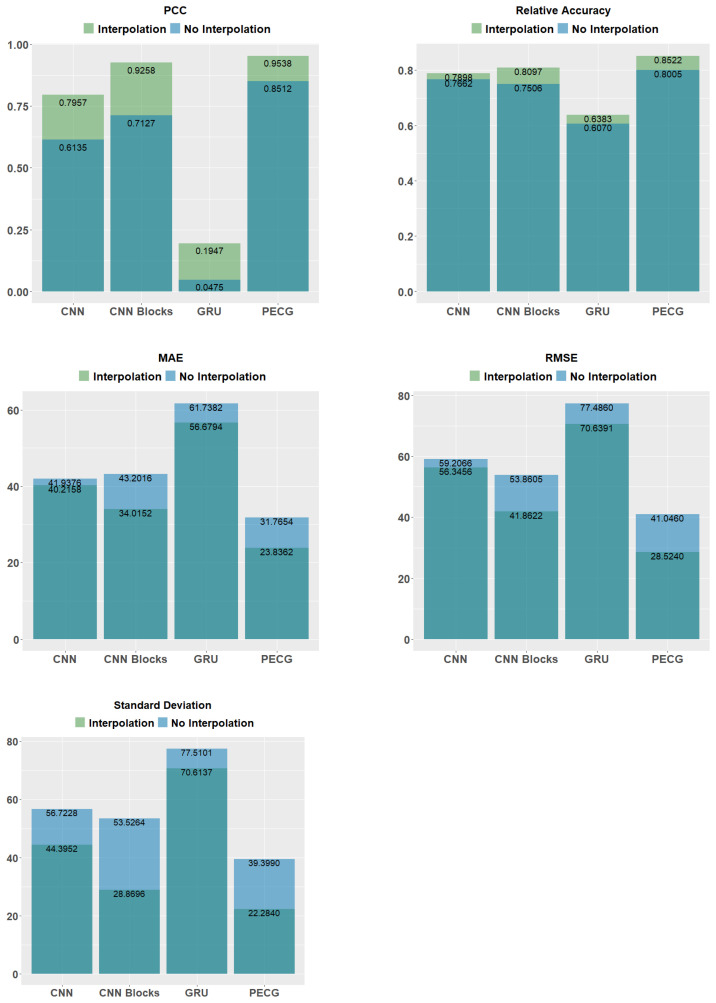
Comparison results with and without interpolation.

In order to further illustrate the effectiveness of the PCHIP method, we take prediction results of four models trained without interpolation processing of missing data on the test set as the baseline. By comparing the predictive outcomes of interpolated and non-interpolated models, it can be inferred that the four models trained on interpolated data exhibit superior performance across all metrics when compared to the models trained on non-interpolated data. Results are shown in Figure 15. It can be seen from the dark blue bars that PECG outperforms other models even when we do not use the PCHIP interpolation method. This demonstrates the superiority of our model architecture. When combined with the PCHIP method, all major metrics of the four models have been improved, further illustrating the effectiveness of the interpolation method we have adopted. The light blue bars in Figure 15 show that by incorporating the PCHIP interpolation method, noteworthy improvements are observed among the evaluated models. Specifically, the standard deviation of CNN Blocks decreases from 53.5264 to 28.8696, representing a significant reduction of approximately 46%. Similarly, the RMSE of PECG decreased from 41.0460 to 28.5240, indicating a substantial decline of approximately 31%. These findings underscore the efficacy of the employed interpolation approach.

### 4.4. Phm 2010 Dataset Results

**Figure 16:**
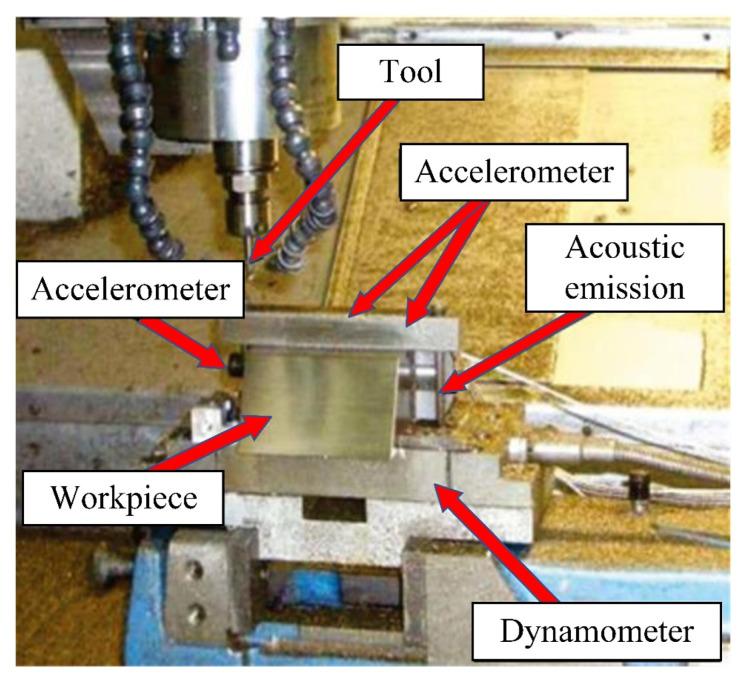
PHM 2010 competition platform [38].

**Figure 17:**
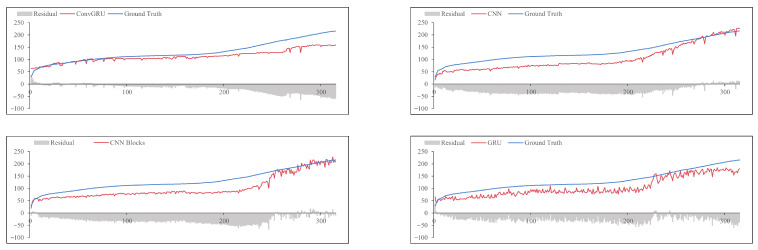
Comparison of results on PHM 2010 dataset.

We validate the performance of the proposed method on the PHM 2010 dataset [37]. The platform of the PHM 2010 competition is shown in Figure 16. The cutting conditions of the dataset remain unchanged, utilizing a 6 mm ball nose tungsten carbide cutter to perform straight tool path cuts on the sidewall of an aluminum alloy blank. The experimental parameters are shown in Table 5. C1 and C4 are used as the training set, while C6 is used as the testing set. The tool scrap standard is 170 μm [18]. The results of different methods are shown below. As is shown in Figure 11, the proposed ConvGRU performs the best when the tool wear value is less than 170 μm. It seems that the other three models predict more accurately than ConvGRU after the wear value exceeds 170 μm. However, in practice, these good performances have no practical significance, because when the tool wear value reaches 170 μm, it is considered to have reached the scrapping criteria and is no longer used. Therefore, the prediction results before reaching the tool scrap criteria are more important. As shown in Figure 17, the proposed ConvGRU model outperforms the other three models significantly in this aspect.

**Figure 18:**
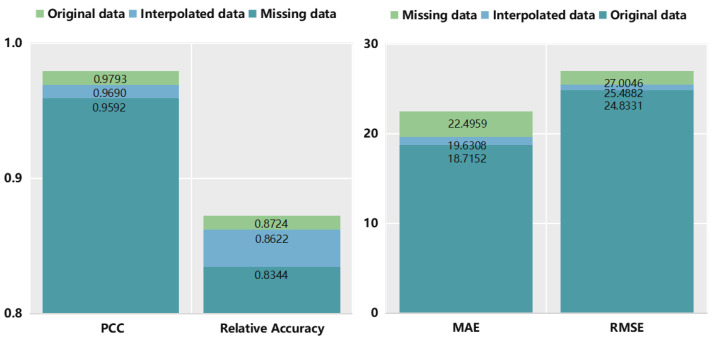
Tool wear prediction performance of PECG on PHM 2010 dataset.

We also prove the effectiveness of data augmentation on the PHM 2010 dataset. Firstly, we randomly selected 20% of the data from the dataset and removed these data points to simulate the scenario where tool wear values are missing in practice. Then, we applied PCHIP interpolation to fill in the missing data, and trained the ConvGRU model on the interpolated dataset. The results are shown in Table 6 and Figure 18. As shown in Table 6, after adopting the data augmentation method of PCHIP interpolation, the prediction performance of the PECG model outperforms the simple ConvGRU model, which is trained without interpolation, in terms of four evaluation metrics: PCC, Relative Accuracy, MAE, and RMSE. Furthermore, the prediction results of PECG are very close to the original dataset, indicating that the proposed PECG model with the data augmentation method PCHIP yields favorable results and exhibits a small gap compared to the results of the model trained by the actual dataset. Therefore, PECG effectively addresses the issue of missing wear data in practical applications while also reducing the cost of multiple wear value measurements.

## 5. Conclusions

In this paper, we introduce an efficient interpolation method known as PCHIP to address the challenge of missing data, specifically in the context of 397 wear prediction. Additionally, we present a novel model named PECG designed for wear prediction tasks.

CNNs possess a remarkable ability to learn hierarchical features from high-dimensional data, rendering them highly effective in capturing informative features for regression tasks. On the other hand, GRUs are known for their efficiency with fewer parameters and faster training speeds. GRUs, as a type of RNN, excel at capturing and modeling long-term dependencies within sequential data, making them particularly suited for time-series regression tasks. Moreover, the inclusion of gating mechanisms in GRUs means they need to learn a limited number of parameters, leading to accelerated training and improved generalization performance when compared to traditional RNNs.

In our approach, we unify CNN and GRU to craft the innovative PECG model. Initially, CNN plays a pivotal role in reducing the input data’s dimensionality and complexity, thus enabling precision in modeling temporal dependencies by the GRU. This fusion capitalizes on the exceptional feature extraction capabilities of CNN and the adeptness of GRU in handling time-series data. Consequently, PECG emerges as an effective predictive model for tool wear prediction, harnessing the strengths of both CNN and GRU.

## Figures and Tables

**Table 1:** Details of model structure.

Layer	Feature Maps	Kernel Size	Parameter Number
CNN-Block_1	128	5	5504
CNN-Block_2-10	128	5	82,304
GRU	384	123	99,072

**Table 2:** Tool working conditions.

Condition	Cutter	Spindle Speed	Feed Rate	Depth of Cut
Condition 1	C1_1 C1_2	2750 rpm	220 mm/min	1.75 mm
Condition 2	C2_1	3000 rpm	200 mm/min	1.75 mm
Condition 3	C3_1	3000 rpm	240 mm/min	1.75 mm
Condition 4	C4_1 …C4_6	3000 rpm	250 mm/min	1.75 mm
Condition 5	C5_1	3250 rpm	275 mm/min	1.75 mm
Condition 6	C6_1	3500 rpm	250 mm/min	1.75 mm
Condition 7	C7_1 …C7_9	3500 rpm	300 mm/min	1.75 mm
Condition 8	C8_1 …C8_7	4500 rpm	400 mm/min	1.5 mm

**Table 3:** Results of interpolation methods.

Methods	PCC	MAE	RMSE	MAPE	Standard Deviation
pchip [29]	**0.9948**	**3.1701**	**4.7902**	0.0191	**4.7682**
cubic spline	0.9942	3.2968	5.0278	**0.0189**	5.0265
spline	0.9932	3.3427	5.4407	0.0206	5.4278
linear	0.9934	3.2514	5.4306	0.0201	5.3822

Bold indicates optimal performance.

**Table 4:** Tool wear performance estimation results of four networks.

Methods	PCC	Relative Accuracy	MAE	RMSE	Standard Deviation
CNN [36]	0.7957	0.7898	40.2158	56.3456	44.3952
CNN Blocks	0.9258	0.8097	34.0152	41.8622	28.8696
GRU	0.1947	0.6383	56.6794	70.6391	70.6137
PECG	**0.9538**	**0.8522**	**23.8362**	**28.5240**	**22.2840**

Bold indicates optimal performance.

**Table 5:** PHM 2010 competition experimental parameters.

Classification	Model/Value	Classification	Value
Machine model	Roders Tech RFM 760	Radial cutting depth	0.125 mm
Workpiece material	Nickel-based superalloy 718	Axial cutting depth	0.2 mm
Tool	3-tooth ball nose milling cutter	Number of sensors	3
Spindle speed	10,400 RPM	Number of sensing channels	7
Feed rate	1555 mm/min	Sampling frequency	50 kHZ

**Table 6:** Tool wear prediction performance of PECG on PHM 2010 dataset.

Data	PCC	Relative Accuracy	MAE	RMSE
Original data	0.9793	0.8724	18.7152	24.8331
Missing data	0.9592	0.8344	22.4959	27.0046
Interpolated data	0.9690	0.8622	19.6308	25.4882

## Data Availability

PHM 2010 is available at https://phmsociety.org/phm_competition/2010-phm-society-conference-data-challenge.

## Abbreviations

The following abbreviations are used in this manuscript:

SAE	Stacked Autoencoder
RUL	Remaining Useful Life
GRU	Gated Recurrent Unit
HMM	Hidden Markov Model
CNN	Convolutional Neural Network
CNN Block	Convolutional Neural Network Block
PCHIP	Piecewise Cubic Hermite Interpolating Polynomial
PECG	PCHIP-Enhanced ConvGRU
PCC	Pearson’s Correlation Coefficient
CNC machine	Computerized Numerical Control Machine
MGRU	Multi-head gated recurrent unit
PCA	Principal component analysis
ANN	Artificial Neural Network
SVM	Support Vector Machine
GPR	Gaussian Process Regression
RNN	Recurrent Neural Network
DNN	Deep Neural Network
ReLU	Rectified Linear Unit
EMA	Exponential Moving Average
MAE	Mean Absolute Error
RMSE	Root Mean Square Error
MAPE	Mean Absolute Percentage Error
